# Evaluating physician associate students’ perceptions of an online team-based learning session on stroke medicine

**DOI:** 10.1136/bmjoq-2024-002966

**Published:** 2025-03-22

**Authors:** Basaam Aweid, Allison Wiseman, Anna Russell, Anjaly Mirchandani, Natalie Parnis, Preman Rajalingam

**Affiliations:** 1 The Hillingdon Hospitals NHS Foundation Trust, Uxbridge, UK; 2 Brunel University London College of Health Medicine and Life Sciences, London, UK; 3 Nanyang Technological University, Singapore

**Keywords:** Clinical Decision-Making, Diagnosis, Teamwork

## Abstract

**Background and aims:**

Team-based learning (TBL) is an effective, active learning strategy that has been validated and used in medical schools. It consists of three phases; preparation, readiness assurance tests and application exercise. It follows a ‘flipped classroom’ model where assessment takes place at the beginning and encourages team discussions that emulate clinical practice. TBL has been used in medical education; however, there is a lack of literature on its use specifically in physician associate (PA) education. We therefore explored the perceptions of a Stroke TBL session among PA students in a UK PA Programme.

**Methods:**

The study took place during the COVID-19 pandemic; therefore, TBL was implemented virtually using online video conferencing platforms. The students’ perceptions were then analysed using anonymous online questionnaires sent to them shortly after the session. The questionnaire included specific questions comparing TBL to other teaching methods such as problem-based learning (PBL).

**Results:**

Overall, the students felt that TBL was an effective teaching method that was better than other methods such as lectures and PBL.

**Conclusions:**

This was a small study of a single TBL session that provided rich qualitative data around students’ perceptions. It is a good foundation for developing TBL further in UK PA Programmes. We encourage further use of this strategy with further studies in this area.

**WHAT IS ALREADY KNOWN ON THIS TOPIC**

- There are a small number of studies that looked at the effectiveness of TBL in PA student education.
- There is a gap in evidence for the effectiveness of TBL within PA education in the UK where it is a relatively new profession.

**WHAT THIS STUDY ADDS**

- This is one of the first UK studies demonstrating positive student perceptions of a TBL session in a UK PA programme.
- TBL was also compared to other small group teaching such as PBL.

**HOW THIS STUDY MIGHT AFFECT RESEARCH, PRACTICE POLICY**

- This qualitative evaluation of a single session could serve as a basis for larger studies looking at an entire module taught using TBL in a PA programme.

## Introduction

 Team-based learning (TBL) is increasingly promoted as an educational strategy for medical students reflecting the move from didactic teaching methods to pedagogical approaches that promote active learning.^1^ ^2^ ^3^ A recent article has shown that TBL enhances clinical reasoning.^4^ Clinical reasoning can be defined as; ‘A skill, process, or outcome wherein clinicians observe, collect and interpret data to diagnose and treat patients. Clinical reasoning entails both conscious and unconscious cognitive operations interacting with contextual factors such as the patient’s unique circumstances and preferences and the characteristics of the practice environment’.^5^ We feel that efficient teaching of clinical reasoning is even more crucial in an intensive 2-year physician associate (PA) course.

The impact of COVID-19 on student learning demanded alternative methods of learning and their delivery. In the UK, similar to other countries, online learning and blended learning have required educators to review how they engage students in their teaching.^6^ This has required students and educators to adapt to new learning styles focusing on active learning in order to contextualise learning into their practice.^7 8^

TBL was developed by Michaelsen^9^ and can be broken down to the following three phases:^2 9^

1. Preparation: the individual completes assigned work prior to class.
2. Assessment: in-class individual and team readiness assurance tests (iRAT and tRAT).
3. Application: group application exercise based on real-life scenarios.

In acknowledgement of the wide variety of approaches to TBL used in medical education, Haidet *et al*^10^ have developed guides for educators using TBL to facilitate its integration within curriculums. We use this as a basis in how we deliver TBL at Brunel University.

Currently, there is a lack of studies exploring TBL and its application to PA education in the UK. In addition, there is only one study that directly compares TBL with other similar teaching methods such as problem-based learning (PBL).^1^ While this showed that students generally preferred TBL over PBL, more work is needed in this area.

In the USA, a number of studies have evaluated PA students’ perceptions of TBL. These identified that student opinions vary across studies looking at TBL’s utility and impact on learning.^11 12^ Isbell *et al*^13^ identified TBL’s positive impact on PA student performance in gross anatomy. Furthermore, Loftin and West^14^ identified the positive effect TBL had on PA students’ confidence in end-of-life care.

PAs are an emerging profession in the UK, commissioned by the department of health in 2004. The PA profession has received increasing prominence in the UK healthcare workforce in the last few years.^15^ PAs are generalists, trained to the medical model but not qualified as doctors. They are supervised by doctors, take medical histories, undertake physical examinations, order simple tests and will see people with acute and long-term conditions, such as stroke and stroke mimics.^16^ In the USA, PAs are referred to as physician assistants where they have been working since the 1960s.^16^ At Brunel University London, we run the PA programme as a 2-year, postgraduate Masters course.^17^ Students must have an undergraduate degree in a health science in order to apply to PA courses in the UK. After graduating, students need to complete national written and practical examinations run by the Royal College of Physicians (RCP) in the UK. Since December 2024, the General Medical Council (GMC) has become the professional body that regulates PAs in the UK.

**Figure 1:**
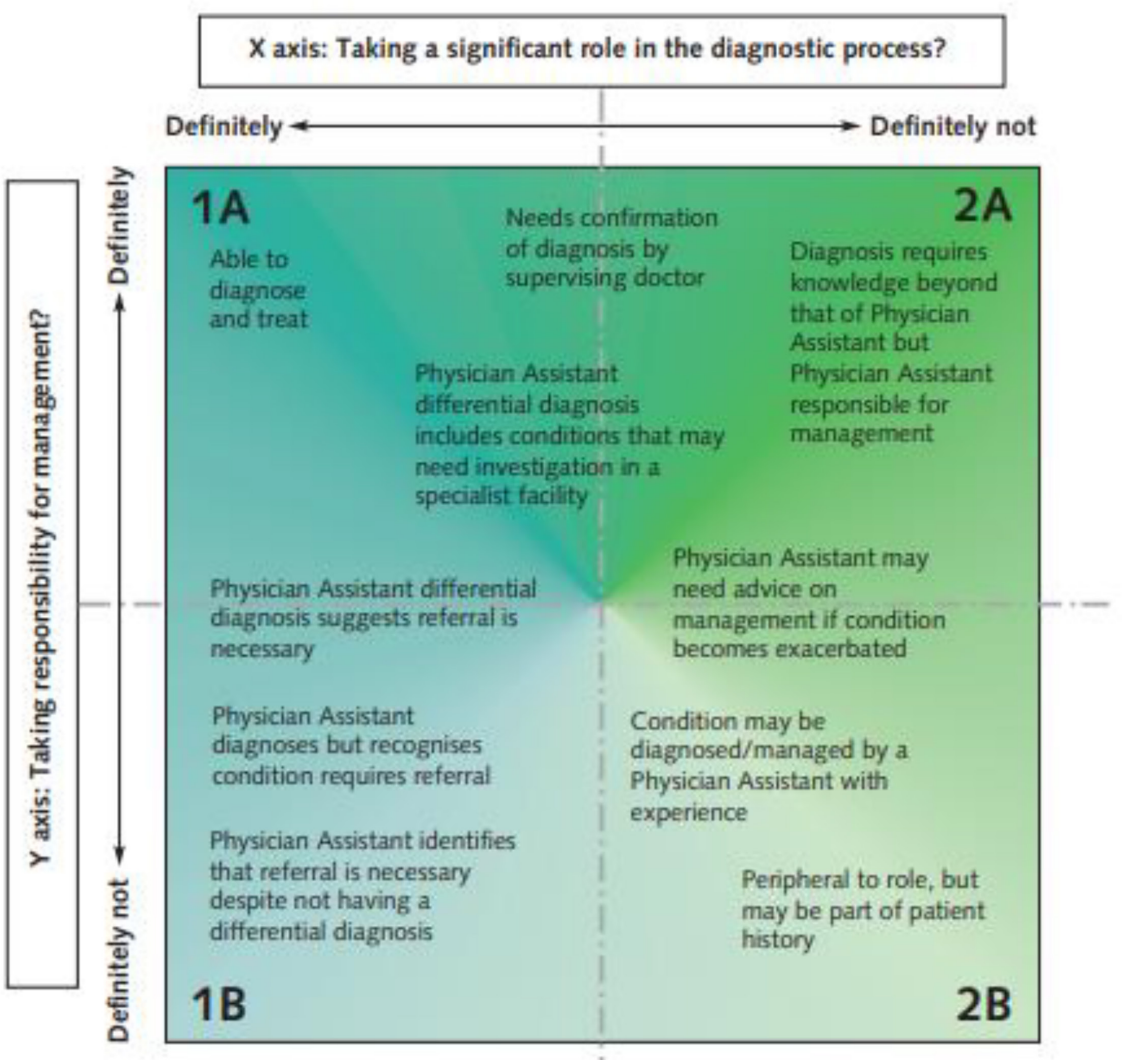
Matrix specification outline. Outline of how different elements of the physician associate curriculum are categorised depending on the depth of clinical knowledge required.

Stroke care is an essential element for PA student education in the UK as part of the competence and curriculum framework for PAs and the Department of Health Matrix of Clinical Conditions.^18^ The Matrix has four categories of conditions (see figure 1) that students must study during their programme. Stroke and stroke type symptoms are common conditions that PA students, similar to other healthcare students, will encounter in practice and are classified as a ‘1B' conditions. Students must therefore be able to identify stroke as a potential diagnosis, be able to refer appropriately and manage deterioration.^18^ The Matrix applied to our cohort however, the PA curriculum has since been updated by the GMC and PA students now follow a new curriculum.

We wanted to use a Stroke TBL session to explore the perceptions of PA students at Brunel University London. As seen from this figure, there is a focus on clinical reasoning. In medical education, clinical reasoning teaching is highlighted as an important element.^19^ Compared with PBL, TBL may be more effective at promoting this skill as it is a more scalable and consistent teaching method.

## Methods

### Context

Brunel University London runs a 2-year PA Masters Programme. The existing curriculum incorporates PBL as a teaching method during year one of the programme. Convenience sampling was used for this study. Year 2 PA students (n=24) were chosen as they all had a year’s worth of PBL experience to draw comparisons from, when considering their preference for PBL or TBL. This student cohort consisted of 65% females and 35% males. They were postgraduates with an age range between 22 and 30.

In light of the COVID-19 pandemic, the TBL sessions were conducted as remote sessions to maintain social distancing. The online sessions were run using Blackboard Collaborate Ultra and the LAMS (learning activity management system) applications. In April 2021, students were given an introductory session on TBL to familiarise them with the format and online applications used. In May 2021, year 2 students had a mandatory teaching session on the topic of stroke, delivered as an online TBL session.

Students were placed into their original year 1 PBL groups for the online TBL session to keep the group dynamics similar to their PBL experiences. The TBL session was led by the medical director of the PA programme, who is a stroke consultant and was not involved with year 1 PBL sessions. The TBL session took place over 120 min which is the same duration as their PBLs. Students had 1 week to revise the preparation material that was sent to them.

### Design

A voluntary, anonymous, online questionnaire was used to explore year 2 PA students’ perceptions and attitudes towards TBL as a teaching method. This was emailed to the students at the end of the session. Students were informed that participation or non-participation would not affect their course studies or assessments in any way. The questionnaire used dichotomous or categorical questions followed by open questions and ‘white space’. The use of open attitudinal and behavioural questions, asking students to consider what they ‘feel’ or asking about their ‘experience’, facilitated the collection of detailed information for analysis.^20^

A seven-item questionnaire was designed that explored the following study objectives:

1. What were the prior experiences of PA students towards TBL?
2. Do PA students feel that TBL is an effective method of learning?
3. Do PA students prefer TBL to other traditional methods of teaching?

**Figure 2:**
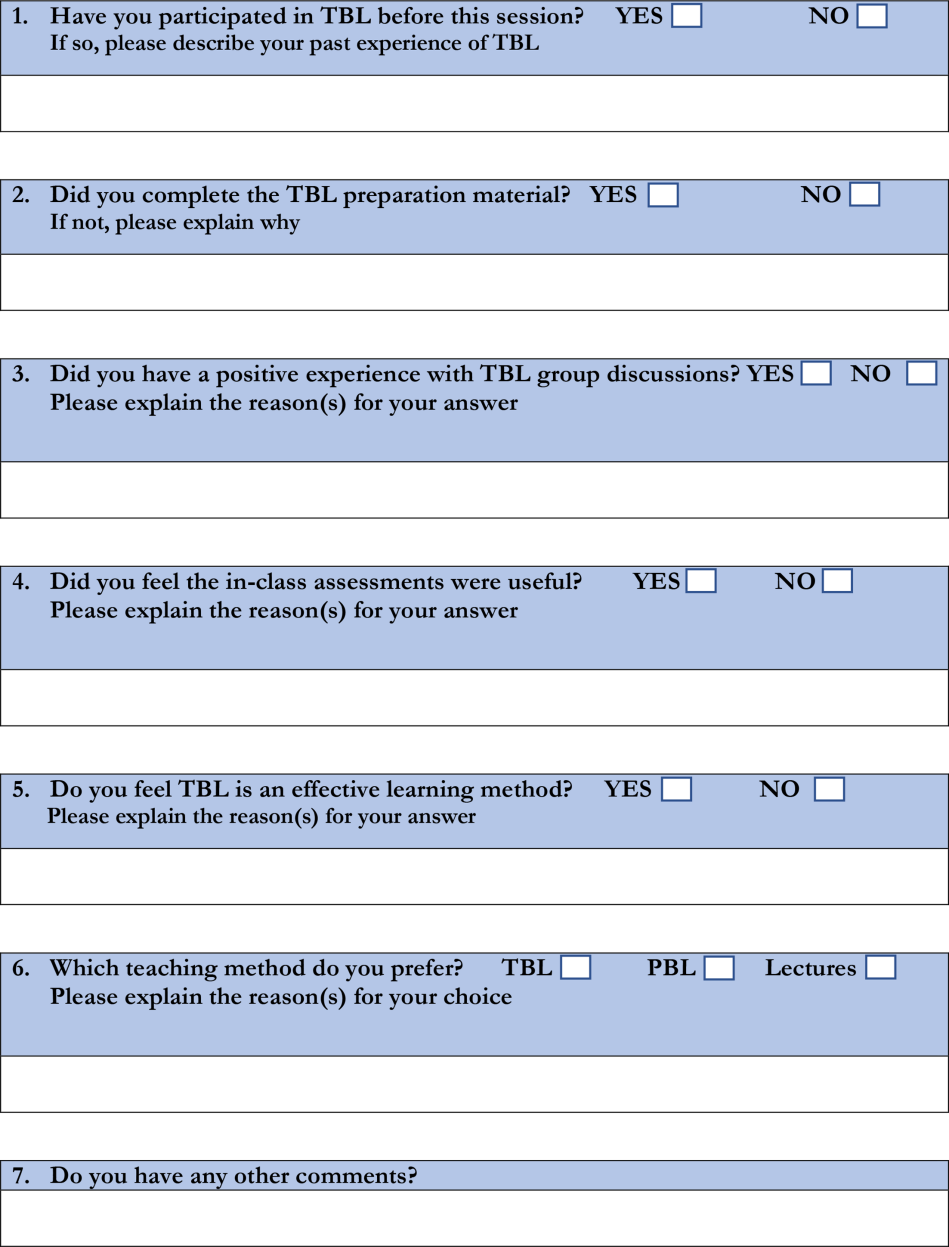
Anonymous online questionnaire. TBL (team-based learning) session feedback. PBL, problem-based learning.

The questionnaire incorporated some of the themes used by Nguyen *et al*^11^ and Patel *et al*^12^ to allow comparison of data. To address an area rarely explored in the literature, a question comparing TBL, PBLs and lectures was also included. Figure 2 illustrates the questionnaire used to evaluate the session.

### Data analysis

**Figure 3:**
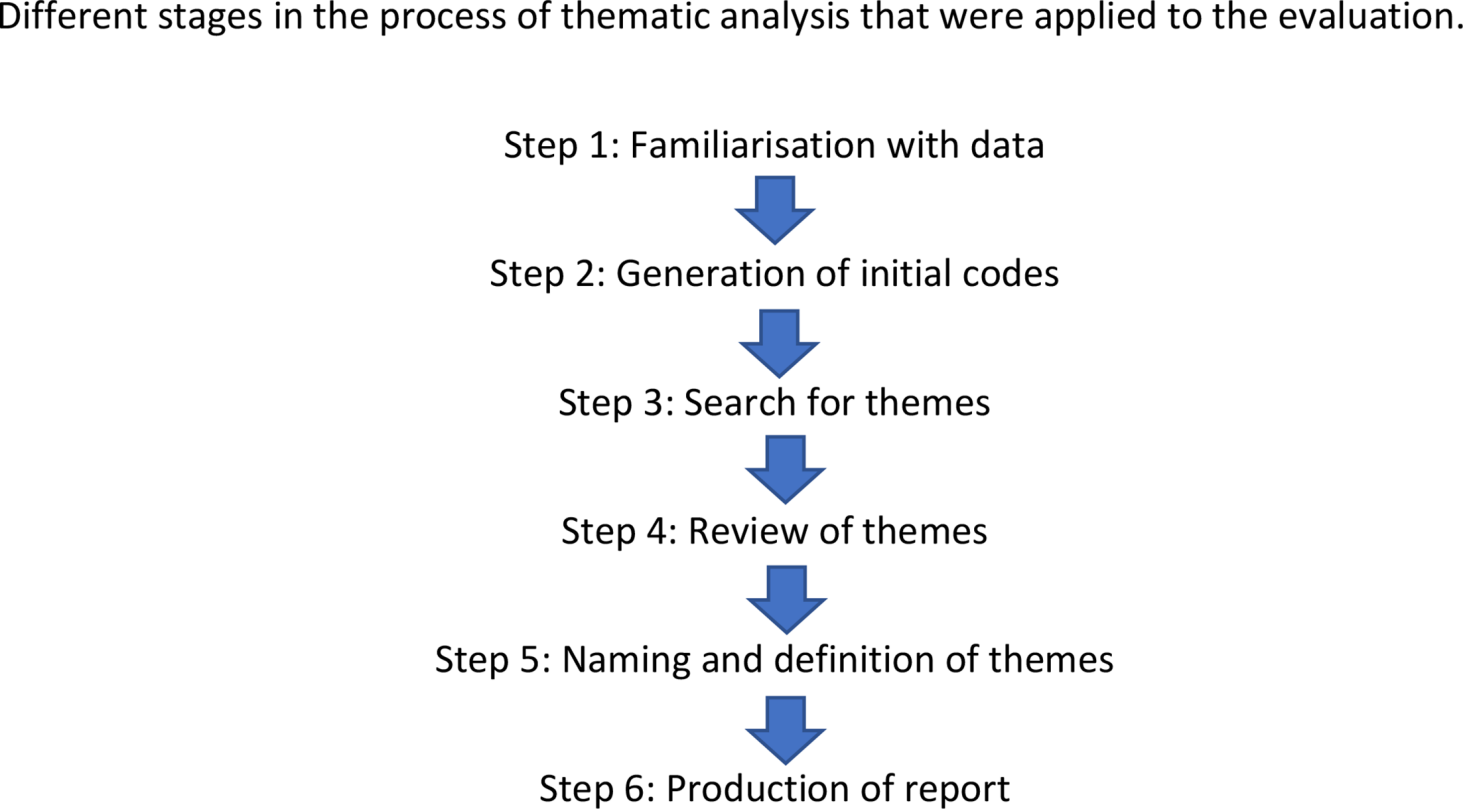
Thematic analysis. Different stages in the process of thematic analysis that were applied to the evaluation.

Thematic analysis was used to analyse the questionnaires.^21^ The steps that make up the analysis are summarised in figure 3. An inductive approach to generation of themes was used and thus themes were drawn from the entire data set. The study was based on a post-positivist epistemology with the analysis focusing on individuals’ meanings and experiences to gain insight into the external reality being investigated.^22^

## Results

**Figure 4:**
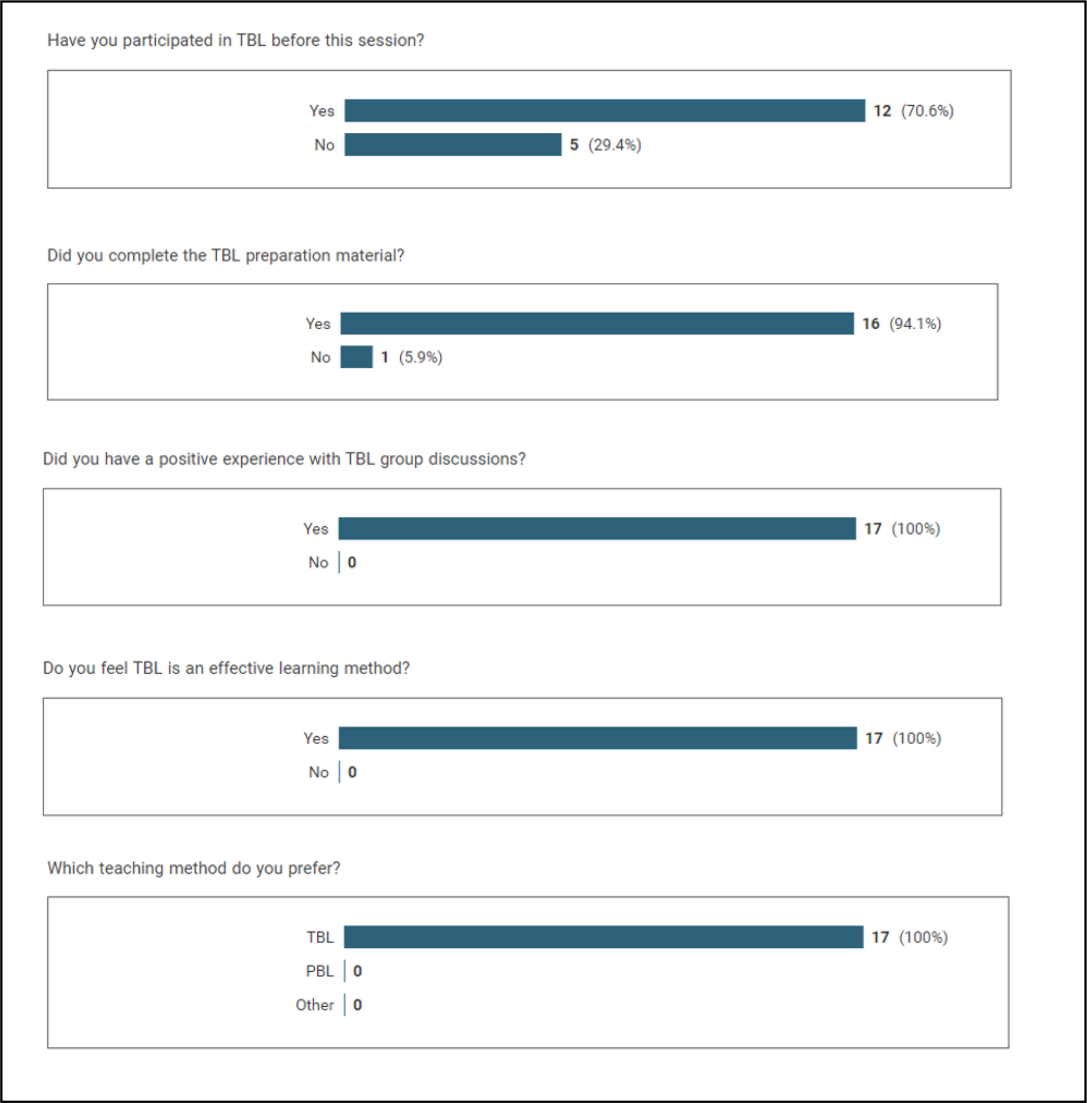
Results of the categorical question from the evaluation. Students were asked to give reasons for each of these answers. PBL, problem-based learning; TBL, team-based learning.

A total of 21 students attended the TBL session on stroke, and of these, 17 (81%) completed a questionnaire regarding the TBL experience. Online supplemental file 1 contains the full results of the survey. Figure 4 outlines the results of the categorical questions.

Students were asked if they had previously participated in TBL and if so to describe their experience. Out of the respondents, 12 (70.6%) had previously participated in TBL. Two students described having participated previously in person, while for seven students, their previous experience was limited to the TBL introductory session prior to the Stroke TBL teaching session.

Student responses to previous experience were overall positive, with students commenting it was ‘interactive and fun’ and that ‘this was very useful and probably the best learning method’. Another student said, ‘initial idea seems good’.

100% of respondents had a positive experience with TBL group discussions. Please see the full responses in box 1.

Box 1 **Reasons for positive experience with team-based learning group discussions. This box contains direct responses from the students when asked to explain their positive experiences**

Very nice to speak to everyone and discuss ideas.

I’m quite an anxious person when it comes to being put on the spot, this helped with the team explaining their reasoning.

All group members participated, interesting to see the different thought processes and explanations to justify each answer.

Group work, everyone participates and inputs their opinions. Help think outside the box.

It is fun, I learn a lot.

It’s helpful when you aren’t confident with a question—other team members can help and give their reasoning for their answer(s).

It was nice to be able to have a healthy debate between small groups.

Good format, allows participants to see their own knowledge then compare with colleagues.

The activities got me thinking and discussing with the team was great as well.

No pressure to get answer wrong, great idea to discuss and learn, for example, when questions are worded difficulty everyone has a different answer.

Nice to work with a team for a change.

Gets you thinking, interactive.

- Allowed better discussions as groups were smaller*.*
- Allowed previously quiet people to get involved without the fear of being called out.
- You learn from others as they may have read, remembered something you didn’t and also explain their answers.

Box 2 **Reasons provided for Question 5; Do you feel team-based learning is an effective learning method? This box provides direct responses from the students when asked to explain their answer to this question**

It gets you thinking outside the box and tricks on how to answer MCQ questions.

100% should be incorporated into teaching.

Able to see others reasoning.

Its very engaging, actively involved and actively thinking*.*

Enjoyed discussing cases alot greater depth and application of knowledge key at this stage in course.

You see other people’s thought processes too.

It gets me thinking.

Encourages engagement and open discussions*.*

It is an active form of learning which helps me to remember information more easily.

Good interaction with own knowledge, consolidated and discussed with colleagues and then addressed by senior*.* Allows students to fully flex their knowledge first and combine it together with colleagues, filling in necessary gaps with the strengths of each other.

Really interactive and enjoyed the SBAs and activities.

But for major topics.

Allows for the combination of various perspectives and highlights your own blindspots.

Group based discussion that engages learning, getting questions right is positive learning exp.

1. It’s easier for people to discuss rather than in lectures where a few people may only talk it allows peer to peer learning and explanation/teaching.

All participants also felt that TBL was an effective learning method (Question 5). Box 2 outlines their responses in this area.

The experience of the stroke TBL session was overwhelmingly positive, with four main themes emerging from the data:

### Teamworking

The students clearly found the teamworking aspect of TBL to be a very valuable experience both in terms of encouraging everyone to participate in the activities as well as learning from other members of the group. Below are excerpts from the feedback around this theme:

All group members participated, interesting to see the different thought processes and explanations to justify each answer.

You learn from others as they may have read, remembered something you didn’t and also explain their answers.

Everyone participates and inputs their opinions.

### Active learning

Students described the TBL session as an effective learning tool. Many students highlighted how interactive the session was, which they found very beneficial as they felt this encouraged active learning. One student commented: ‘its very engaging, actively involved and actively thinking’. TBL also encouraged students to develop their learning, with another student explaining that it ‘gets you thinking outside the box’ and ‘highlights your own blindspots’. Students also identified that TBL allowed greater depth of discussion and helped to facilitate application of their knowledge. One student explained: ‘TBL is better for …what to do in practice*’*

### Inclusivity

As well as enhancing their educational development, students also expressed that TBL provided a supportive environment, particularly for those who are less confident or anxious. Specific comments were as follows:

I’m quite an anxious person when it comes to being put on the spot, this helped with the team explaining their reasoning.

Allowed previously quiet people to get involved without fear of being called out.

### Preference for TBL over PBL as a teaching method

All 17 respondents preferred TBL as a teaching method in comparison to PBL for the reasons cited above as well as due to the interaction involved. Students’ responses are summarised in box 3.

Box 3 **Reasons provided for Question 6; Which teaching method do you prefer?**

More interactive with people from class.

PBL is good for learning a new topic and public speaking but TBL is better for recap and revision of guidelines and what to do in practice.

I liked TBL sessions to learn as it tests your individual knowledge and then allows is to know our peers point of views and thinking strategies.

More group engagement in smaller groups.

PBL got nothing out if it—just copy the internet into a slide adds no understanding or depth not like TBL.

More interactive.

Much more convenient and useful.

More interactive.

TBL more interactive, to the point and allows ability to engage with all students.

PBL takes up a lot of time to present and prep the feedback from the lecturers can be vague and we don’t receive full 100% attention from everyone as there are multiple presentations in a short amount of time.

TBL and PBL would work well together. I’m actively learning in TBL so its better.

Problem based is good but it’s not as multi-faceted as TBL and so you can incorporate PBL in a TBL situation and getting a better outcome.

Only one student commented on the involvement of lecturers with regards to PBL ‘the feedback from lecturers can be vague’, but there were no comments with regards to facilitation within TBL.

Two students expressed that TBL would be better in a face-to-face environment rather than remotely due to technical limitations imposed by virtual sessions.

## Discussion

We have demonstrated that TBL can be successfully used in a UK healthcare course such as our PA Programme. Overall, students found TBL to be a positive teaching experience, indicating a desire for more of their topics to be delivered in this way. The themes of ‘teamworking’ and ‘inclusivity’ demonstrated the unique aspects of TBL. It allowed individuals who may normally be quiet in the classroom to actively engage. This has important implications not only during their course but also for their future professional career. These unique skills may not be practised as well through PBL. Students indicated this by describing TBL as providing more ‘in depth understanding’ and being more ‘multi-faceted’.

For this study, we surveyed second-year PA students. During their second year, students spend most of their time on clinical placements. This is a much more practical year involving interaction with clinicians and patients. First-year students spend the majority of their time in didactic teaching and PBL. The results may have been different if this study had been undertaken with the first-year students. We also carefully selected the TBL teams to make sure the students were familiar with one another. Both these factors may have made the session more effective.

### Limitations

The limitations of the study include a relatively small sample size and its restriction to only one TBL session. Detailed student demographics were not collected, specifically the age of each respondent could have been relevant in a postgraduate course where a student’s maturity may have influenced their perception of TBL. As with the introduction of any new strategy, it may have been subject to the ‘novelty effect’.^23^ It would need to be demonstrated that these positive attitudes are consistent after multiple TBL sessions for this cohort. At that point, we could review the impact of TBL on a whole module or term. This study was qualitative with an evaluation of the students’ subjective impressions. A quantitative assessment of the student’s knowledge could have been made by comparing the individual (iRAT) and team (tRAT) scores to see if there was a significant improvement.

### Future research

It is difficult to conclude whether TBL can completely replace all other teaching methods. Further research should be undertaken to explore if TBL meets different learners’ needs. It would also be important to evaluate the effect of TBL on graduates who have been taught using this strategy. Only then would we be able to conclude if TBL can successfully teach clinical reasoning, one of the areas we were also interested in exploring.

To understand the sustainability of this approach, it would be essential to gather academic staff feedback on TBL. An effective teaching method needs to also be practical within the available resources of a department.

Our survey focused on ‘white space’ questions that allowed for the analysis of rich qualitative data. In a future project with a larger sample size, Likert-scale questions would help quantify students’ feedback. In addition, results from summative examinations of cohorts taught traditionally could be compared with the results of students who were taught using TBL. Our study lays the ground for such mixed-method research in a UK PA programme, similar to what previous authors have done in the USA.^11 12^

As a next step, we plan to run a whole module using TBL for our next PA cohort. We would then assess the PA student perceptions and performance in a mixed methods study as explained above.

Finally, aside from being one of the only studies exploring TBL in PA students in the UK, this study was undertaken on TBL delivered online. In this context, it successfully demonstrated an effective teaching strategy that can be implemented in the middle of a pandemic or other situations where face-to-face teaching may be challenging.

## Conclusions

TBL therefore represents an improvement in the quality of teaching by offering a flexible teaching environment in addition to inclusive, active and effective team discussions that may be better than PBL.

## Supplementary material

10.1136/bmjoq-2024-002966 online supplemental file 1

## Data Availability

Data are available upon reasonable request.
