# Supplementary material for: Evaluating physician associate students’ perceptions of an online team-based learning session on stroke medicine
Source: BMJ Open Qual. 2025 Mar 22;14(1):e002966. doi: 10.1136/bmjoq-2024-002966 (PMC11931953; doi:10.1136/bmjoq-2024-002966)
Supplement: online supplemental file 1 [file bmjoq-14-1-s001.pdf]

What are the Perceptions and attitudes of Physician Associate (PA) students towards Team Based Learning (TBL) as a teaching method?

Showing 17 of 17 responses

Showing **all** responses

Showing **all** questions

Response rate: 17%

1 I have read the Participant Information Sheet included with this questionnaire

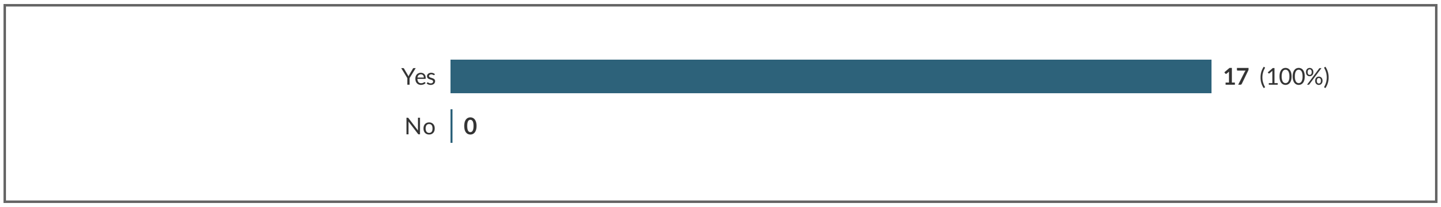

1.a I am over the age of 18

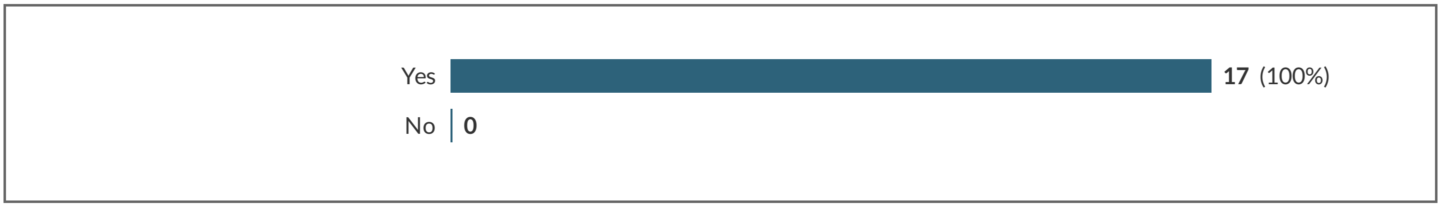

1.b I understand that no personal identifying data is collected in this study, therefore I know that once I have submitted my answers I am unable to withdraw my data from the study

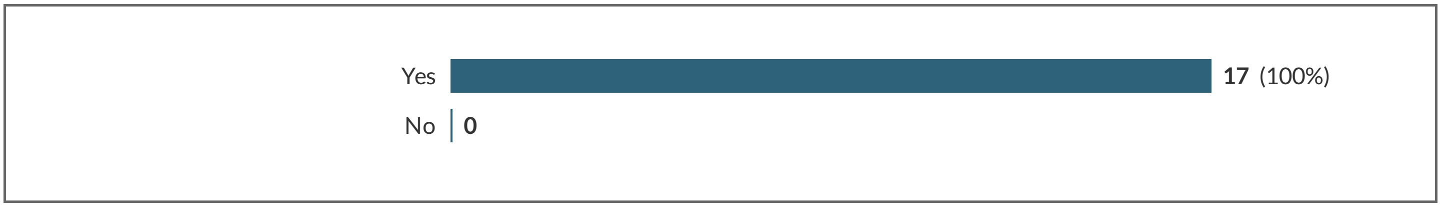

1.c I agree that my data can be anonymised, stored and used in future research in line with University's data retention policies

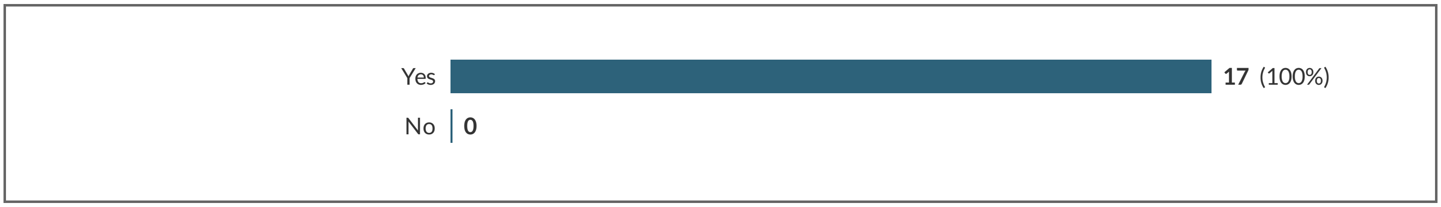

1.d I agree to take part in this study

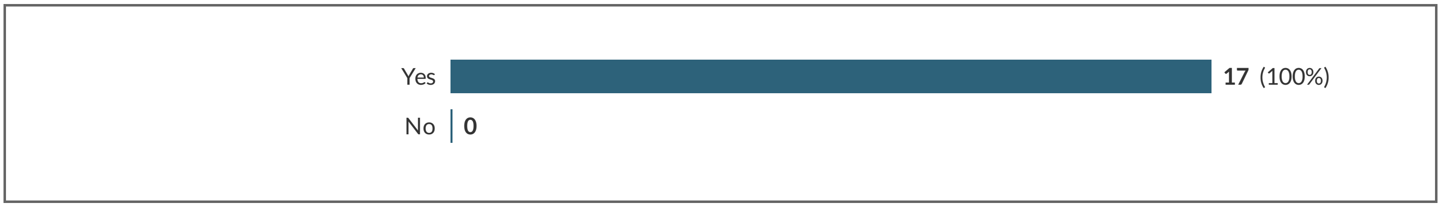

2 Have you participated in TBL before this session?

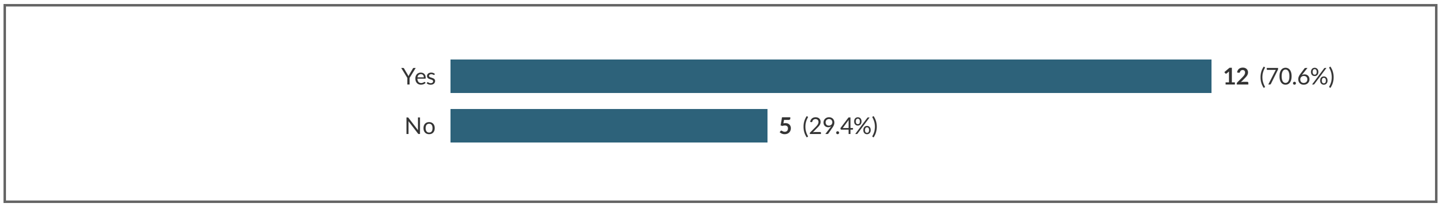

2.a If so, please describe your past experience of TBL

Showing all 11 responses	
previously in person	737907-737898-77557298
good with mcq style questions to revise from and discuss views with the team	737907-737898-77557294
one week ago trial session - not relevant to course so did not enjoy it	737907-737898-77557325
introductory session	737907-737898-77557297
The first session we had	737907-737898-77557296
online - despite this was very useful and probably the best learning method.	737907-737898-77557305
had one session before, was interactive and fun	737907-737898-77557333
Yes the trial session last week	737907-737898-77557322
In personal through undergraduate degree as a summative assessment.	737907-737898-77557402
Intro session last week	737907-737898-77557530
last week, introduction but the initial idea seems good	737907-737898-77557315

3 Did you complete the TBL preparation material?

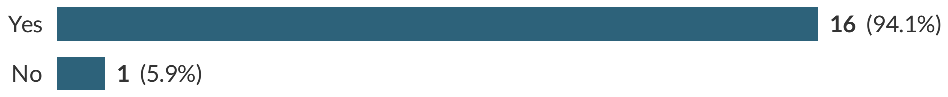

3.a If not, please explain why

**Showing 1 response**	
Busy doing various other work and felt that I have an alright comprehension of the relevant material already.	737907-737898-77557402

4 Did you have a positive experience with TBL group discussions?

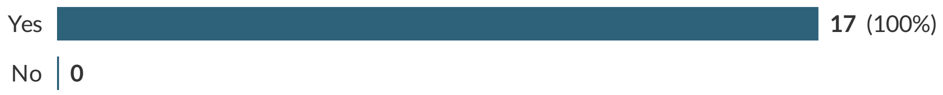

4.a Please explain the reason(s) for your answer

Showing all 13 responses	
Very nice to speak to everyone and discuss ideas	737907-737898-77557302
im quite an anxious person when it comes to being put on the spot, this helped with the team explaining their reasoning	737907-737898-77557298
all group members participated, interesting to see the different thought processes and explanations to justify each answer	737907-737898-77557295
Group work, everyone participates and inputs their opinions. Help think outside the box.	737907-737898-77557338
It is fun, I learn a lot	737907-737898-77557372
it's helpful when you aren't confident with a question - other team members can help and give their reasoning for their answer(s)	737907-737898-77557297
It was nice to be able to have a healthy debate between small groups	737907-737898-77557296
good format, allows participants to see their own knowledge then compare with colleagues	737907-737898-77557305
The activities got me thinking and discussing with the team was great as well	737907-737898-77557333
no pressure to get answer wrong<br>great idea to discuss and learn for example when questions are worded difficulty everyone has a different answer	737907-737898-77557322
Nice to work with a team for a change.	737907-737898-77557402
Gets you thinking, interactive	737907-737898-77557530
1. allowed better discussions as groups were smaller.<br>2. allowed previously quiet people to get involved without the fear of being called out<br>3. you learn from others as they may have read, remembered something you didn't and also explain their answers	737907-737898-77557315

5 Do you feel TBL is an effective learning method?

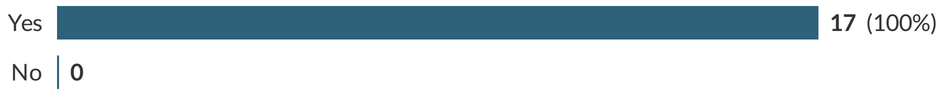

5.a Please explain the reason(s) for your answer

Showing all 15 responses	
it gets you thinking outside the box and tricks on how to answer mcq questions	737907-737898-77557300
100% should be incorporated into teaching	737907-737898-77557302
able to see others reasoning	737907-737898-77557298
its very engaging, actively involved and actively thinking	737907-737898-77557295
enjoyed discussing cases alot greater depth and application of knowledge key at this stage in course	737907-737898-77557325
You see other people's thought processes too	737907-737898-77557338
It gets me thinking	737907-737898-77557372
encourages engagement and open discussions	737907-737898-77557297
It is an active form of learning which helps me to remember information more easily.	737907-737898-77557296
good interaction with own knowledge, consolidated and discussed with colleagues and then addressed by senior. Allows students to fully flex their knowledge first and combine it together with colleagues, filling in neceesary gaps with the strengths of each other	737907-737898-77557305
really interactive and enjoyed the SBAs and activities	737907-737898-77557333
but for major topics	737907-737898-77557322
Allows for the combination of various perspectives and highlights your own blindspots.	737907-737898-77557402
Group based discussion that engages learning, getting questions right is positive learning exp	737907-737898-77557530
1. it's easier for people to discuss rather than in lectures where a few people may only talk<br>it allows peer to peer learning and explanation/teaching	737907-737898-77557315

6 Which teaching method do you prefer?

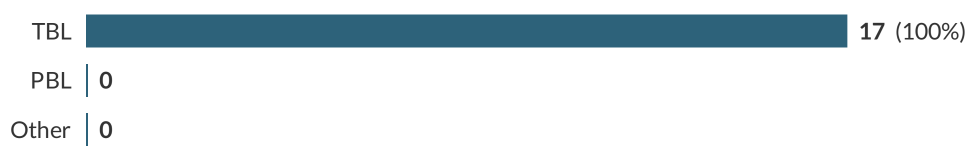

6.a Please explain the reason(s) for your choice

Showing all 13 responses	
More interactive with people from class	737907-737898-77557302
PBL is good for learning a new topic and public speaking but TBL is better for recap and revision of guidelines and what to do in practice	737907-737898-77557294
I liked TBL sessions to learn as it tests your individual knowledge and then allows is to know our peers point of views and thinking strategies.	737907-737898-77557301
more group engagement in smaller groups	737907-737898-77557295
pbl got nothing out if it - just copy the internet into a slide adds no understanding or depth not like TBL	737907-737898-77557325
More interactive	737907-737898-77557372
much more convenient and useful	737907-737898-77557297
more interactive	737907-737898-77557296
TBL more interactive, to the point and allows ability to engage with all students	737907-737898-77557305
more engaging and different	737907-737898-77557333
PBL takes up a lot of time to present and prep<br>the feedback from the lecturers can be vague and we dont receive full 100% attention from everyone as there are multiple presentations in a short amount of time	737907-737898-77557322
TBL and PBL would work well together. I'm actively learning in TBL so its better	737907-737898-77557530
problem based is good but it's not as multi-faceted as TBL and so you can incorporate PBL in a TBL situation and getting a better outcome	737907-737898-77557315

7 Do you have any other comments?

Showing all 9 responses	
Really effective method to learn and teach others	737907-737898-77557302
Would prefer shorter TBL sessions with other forms of teaching mixed in such as some lecture some TBL and some PBL. On its own its hard to keep focus.	737907-737898-77557301
IF ONly we had TBL before this session it would have made such a difference	737907-737898-77557325
None at the moment	737907-737898-77557372
None	737907-737898-77557296
good! Please incorporate TBL into future PA teaching	737907-737898-77557305
great!	737907-737898-77557322
Would be better in person due to the limitations imposed by latency.	737907-737898-77557402
i like the quizzes as it helps with revision and just to re-affirm learning<br>2. i think when it would be better in a F2F environment as technical issues can hinder some individuals experience but even remotely i prefer this method as it makes you think	737907-737898-77557315
